# Adverse Psychosexual Impact Related to the Treatment of Genital Warts and Cervical Intraepithelial Neoplasia

**DOI:** 10.1155/2013/264093

**Published:** 2013-03-24

**Authors:** Adriana Bittencourt Campaner, Nelson Vespa Junior, Paulo César Giraldo, Mauro Romero Leal Passos

**Affiliations:** ^1^Department of Obstetrics and Gynecology, School of Medicine of Santa Casa de São Paulo, Avenida Rebouças 1511, ap. 142, Jd. América, 05401-200 São Paulo, SP, Brazil; ^2^Department of Obstetrics and Gynecology, Federal University of Campinas, Brazil; ^3^Department of Obstetrics and Gynecology, Federal Fluminense University, Rio de Janeiro, RJ, Brazil

## Abstract

*Objective*. To compare the psychosexual impact related to the treatment of genital warts and cervical intraepithelial neoplasia (CIN) in women. *Methods*. 75 patients presenting with HPV-induced genital lesions, belonging to one of two patient groups, were included in the study: 29 individuals with genital warts (GWs) and 46 individuals with CIN grades 2 or 3 (CIN 2/3). Initially, medical charts of each woman were examined for extraction of data on the type of HPV-induced infection and treatment administered. Subjects were interviewed to collect sociodemographic data as well as personal, gynecologic, obstetric, and sexual history. After this initial anamnesis, the Sexual Quotient-Female Version (SQ-F) questionnaire was applied to assess sexual function. After application of the questionnaire, patients answered specific questions produced by the researchers, aimed at assessing the impact of the disease and its treatment on their sexual lives. *Results*. It is noteworthy that patients with CIN 2/3 had statistically similar classification of sexual quotient to patients with GWs (*P* = 0.115). However, patients with GWs more frequently gave positive answers to the specific questions compared to patients with CIN 2/3. *Conclusion*. Based on these findings, it is clear that GWs have a greater impact on sexual behavior compared to CIN 2/3.

## 1. Introduction

The term sexually transmitted diseases (STDs) is used to refer to a variety of clinical syndromes caused by a wide variety of pathogenic microorganisms that can be acquired and transmitted through sexual activity [1]. Human papillomavirus (HPV) infection is the most common cause of STDs in humans worldwide. Epidemiological studies suggest that about 80% of women will have acquired genital HPV by age 50, which makes HPV infection the norm rather than the exception. Age-standardized HPV prevalence worldwide has been shown to vary, nearly 20 times between populations [2, 3].

HPV is a wide family of DNA viruses that may cause benign skin and mucosal tumors (warts) or premalignant/malignant disease in different organs. To date, over 100 different viral types have been identified, and about one-third of these infect epithelial cells in the genital tract. Nononcogenic or low-risk HPV types (mainly HPV types 6 and 11) are the cause of genital warts (GWs). Persistent oncogenic HPV infection, or high-risk HPV types, is the strongest risk factor for the development of precancers and cancers of the anogenital tract [4, 5].

Treatment for clinical sequelae such as GWs includes a wide range of interventions (ablative and cytodestructive; nonsurgical treatments/cytotoxic agents), according to the type and site of lesions, extension, and severity and consists of removing the problematic cells. CIN 2/3 arise almost exclusively at the squamocolumnar junction, and excisional therapies such as loop excision of the transformation zone are highly effective. These mentioned methods consume significant health care resources and are costly. However, some costs are difficult to estimate such as personal distress, psychological comorbidities, and negative sexual outcomes. Early and late recurrences of HPV infection and related pathologies are frequent [6]. 

The literature on HPVs is substantial and increasing. The main areas of research include the virological characteristics of HPV, epidemiology, medical and oncological impact of the infection and related diseases, prevention strategies, diagnosis, pap smear screening, HPV molecular biology, and the potential role of vaccines. However, research investigating the relationship between HPV infections and sexual dysfunctions in women is limited. However, HPV-related diseases may have a significant impact on women's sexuality. It is only in recent years that research in this area has increased. This has occurred in parallel with the growing rate of infections and consequent psychosocial burden [1, 7]. 

HPV-related diseases may have a significant impact on women's sexuality because (a) it is an STD particularly affecting the vulva and the uterine cervix. For biological, emotional, and symbolic reasons, they are the key organs for women's eroticism; (b) it may contribute to vulvodynia and sexual pain disorders, associated with and/or consequent to vulvar treatment; (c) it is a potentially oncogenic disease, which may convey a more serious threat for the woman's genital and general health, specifically increasing fear and anxiety [7]. So, in the present study, we aimed to evaluate the impact of HPV infections and treatment for genital warts and cervical intraepithelial neoplasia (CIN) on women's psychosexual health.

## 2. Material and Methods

An observational, cross-sectional, comparative study was conducted at the Clinic of Lower Genital Tract Diseases and Colposcopy of the Department of Obstetrics and Gynecology of the Irmandade da Santa Casa de Misericórdia de São Paulo Hospital (Brazil), between January 2011 and January 2012. The current study was approved by the Scientific Committee of the Department and the Research Ethics Committee of the institution. A total of 75 patients previously treated for HPV-related genital lesions were included in the study. Prior to inclusion in the study, all participants signed an informed consent form. We offered to all patients who attended our clinic, during the study period, the opportunity to participate in the study; however, the women should meet the criteria for inclusion/exclusion.

### 2.1. Inclusion Criteria

Patients presenting with HPV-induced genital lesions previously submitted to the respective treatment and belonging to one of two patient groups were included in the study: (a) individuals with genital warts (GWs) and (b) individuals with cervical intraepithelial neoplasia grades 2 or 3 (CIN 2/3).

The sample size was based on the calculation of sample size for prevalence, so it was adopted a 5% error and a significance level of 10%. For this calculation, a total number of 50 patients, 25 of CIN and 25 of GWs were found. However, we decided to include all patients who attended to the consultations in the mentioned period and that met the inclusion and exclusion criteria, with the exception of a patient with GWs who refused to answer the questionnaire. In our service, the number of cases of CIN is greater than the number of cases of GWS. So, since we obtained a larger number of patients in each group, higher than expected, the statistician advised us that we could keep this number of patients, even though the number of patients in each group was unequal.

Cases presenting with CIN 1 were not included given that this form of the disease is not considered a cervical cancer precursor lesion, requiring only rigorous followup. It was decided not to employ a control group, since one disease was regarded as a control for the other; it should be noted that the validated questionnaire used in this study had been previously applied to a general population, that is, a control group.

### 2.2. Exclusion Criteria

Patients with newly diagnosed HPV-induced disease and subjects currently undergoing treatment or treated within the last six months were excluded. Immunosuppressed women (for disease or iatrogenically) were excluded, as were those who declined to take part in the interview or were not yet aware of their diagnosis. To be eligible for inclusion, patients could not present with GWs and CIN 2/3 concomitantly. We have excluded a total of 30 patients for CIN group and 9 for GWs group, who were treated less than 6 months from the date of the interview.

### 2.3. Methods

Initially, medical charts of each woman were examined for extraction of data on the type of HPV-induced infection and treatment administered. Patients were approached by the principal investigator and invited to take part in the study while awaiting their consultations. For the present study, we emphasize the performance of sample calculation that resulted in a total of 60 patients. After signing an informed consent form, subjects were interviewed to collect sociodemographic data as well as personal, gynecologic, obstetric, and sexual history. After this initial anamnesis, the Sexual Quotient-Female Version (SQ-F) questionnaire was applied.

### 2.4. The Sexual Quotient-Female Version (SQ-F) Questionnaire

This questionnaire was devised under the Sexuality Studies Program of the Institute of Psychiatry of the Clínicas Hospital of the University of São Paulo School of Medicine (Brazil). The SQ-F instrument is useful for investigating female sexual activity and has been designed especially for use in the Brazilian population. The questionnaire can also be used to stratify patients into clinical or observational study groups and to measure the efficacy of interventions aimed at treating sexual dysfunctions in women. This version of the instrument was devised and validated by Abdo in 2006 [8] and comprises ten questions investigating each phase of the sexual response cycle and other aspects of sexual activity. Responses should be based on the last six months of the woman's sexual life and scored as follows: 0: never; 1: rarely; 2: sometimes; 3: approximately 50% of the time; 4: most of the time; 5: always. The instrument includes the following domains: desire and sexual interest (questions 1, 2, and 8); foreplay (question 3); excitation of the woman and harmonious interaction with partner (questions 4 and 5); comfort in sexual intercourse (questions 6 and 7); orgasm and sexual satisfaction (questions 9 and 10). 

Thus, the SQ-F questionnaire can screen for specific dysfunctions of desire, excitation of the woman, orgasm, dyspareunia, and vaginismus. Sexual activity is also assessed for each separate domain through individual analysis of the questions pertinent to the different aspects investigated. Global performance/sexual satisfaction is assessed based on a final score calculated by summing the scores on the ten questions and multiplying the result by two. The performance/satisfaction results are interpreted on a scale from 0 (minimum) to 100 (maximum) points distributed as follows: 82–100 points (good to excellent); 62–80 (regular to good); 42–60 (unfavorable to regular); 22–40 (poor to unfavorable); 0–20 (null to poor). After application of the questionnaire, patients answered specific questions produced by the researchers aimed at assessing the impact of the disease and its treatment on their sexual lives.

### 2.5. Statistical Analysis

For the statistical analysis of results, the chi-square test, Student's *t*-test and Mann-Whitney test were applied as appropriate. Levels ≤ 0.05 were considered significant.

## 3. Results

In this study involving 75 patients, 46 (61.3%) had a histological diagnosis of CIN 2/3 and 29 (38.7%) of GWs. Mean age of the whole patient group was 37.7 ± 15.1 years, and range was 16 to 79 years. In terms of schooling level, four (5.3%) patients were illiterate, 29 (38.7%) had 1st grade education (concluded or incomplete), 23 had 2nd grade education (30.7%), (concluded or incomplete) and 19 had 2nd grade education (25.3%) had 3rd grade (concluded or incomplete) education. Marital status of the 75 patients was distributed as follows: 22 (29.3%) married, 9 (12.0%) stable union, 9 (12.0%) separated, 34 (45.3%) single, and one widow (1.3%).

The group of patients with cervical lesions CIN 2/3 showed a mean age of 43.7 ± 14.8 years, ranging from 18 to 79 years. Of these, 16 (34.8%) were married, 7 (15.2%) had stable union, 7 (15.2%) were separated, 15 (32.6%) were single, and only one (2.2%) patient was a widow. Already, the patients from group with genital warts had a mean age of 28.3 ± 10.1 years, ranging from 16 to 55 years. Six (20.7%) patients were married, two (6.9%) had stable union, two (6.9%) were separated, and 19 (65.5%) were single. Only one patient in the GWs group was in the menopause; the corresponding value for the group of patients with CIN was 13.

The mean number of pregnancies observed among the 75 patients was 2.7 ± 3.2, ranging from zero to 15 gestations. The number of childbirths was, on average, 2.4 ± 3.0, ranging from zero to 15 births. The mean number of abortions was 0.3 ± 0.7, ranging from zero to 3 abortions. Mean age at commencement of sexual activity among the 74 subjects was 17.8 ± 3.1 years, ranging from 11 to 28 years. The mean number of partners of the 74 patients was 3.7 ± 3.9, ranging from 1 to 30 partners. It should be noted that information on age of commencement of sexual activity and number of partners was not available for one patient. Of the total 75 patients, 48 (64.0%) reported having permanent partners at the time of interview. Mean time in the current relationship, as reported by the 45 patients with partners, was 8.3 ± 11.1 years, ranging from 1 to 60 years. The remaining subjects had no fixed partner at the time of interview. 

Among the 46 patients in the CIN group, the mean number of pregnancies was 4.0 ± 3.5, ranging from none to 15 pregnancies. On average, the number of births was 3.5 ± 3.3, ranging from none to 15 deliveries. The mean age at onset of sexual activity of 45 patients was 17.4 ± 2.7 years, ranging from 11 to 26 years. The average number of partners, reported by 45 patients in this group, was 3.1 ± 2.5, ranging from 1 to 15 partners. Among the 29 patients in the GWs group, the average number of pregnancies was 0.7 ± 0.8, ranging from none to 3 pregnancies. On average, the number of births was 0.6 ± 0.7, ranging from none to two births. The mean age at onset of sexual activity among the patients in this group was 18.2 ± 3.8 years, ranging from 12 to 28 years. The average number of partners reported by the patients in this group was 4.6 ± 5.3, ranging from 1 to 30 partners.

Family income reported by 73 patients revealed that 47 (64.4%) had a low monthly income while 26 (35.6%) had a median monthly income. Only 13 (17.3%) patients were smokers, with average time smoking of 18.2 ± 13.1 years and range of 1 to 56 years. The mean number of cigarettes consumed daily by smokers was 13.1 ± 5.9, ranging from 3 to 20 cigarettes per day. Of the total, 11 patients (14.7%) were former smokers, having discontinued the habit an average of 10.9 years prior, with a range of 1 to 50 years and standard deviation of 14.2 years.

At the time of interview, 45 (60.0%) patients reported use of contraceptive methods, namely, male condoms (17 patients), oral hormonal birth control (20 patients), injectable birth control (4 patients), tubal sterilization (3 patients), and IUD (1 patient) methods. Of the 74 patients assessed, 36 (48.6%) reported use of condoms in the current relationship while 38 (51.4%) did not use condoms. Fourteen (19.2%) out of the 73 patients reported knowing about the infidelity of their current partner. With regard to patients, five (6.8%) reported having been unfaithful to their current partners. Of the 74 patients, four (5.4%) reported having a stable or sporadic extramarital affair.

The inferential results indicated that both groups have statistically equal profiles for marital status (*P* = 0.079; chi-square test), smoking habit (*P* = 0.058; chi-square test), age at commencement of sexual activity (*P* = 0.290; Student's *t*-test), number of partners (*P* = 0.063; Mann-Whitney test), and use of contraceptive method (*P* = 0.081; chi-square test). Patients in the CIN 2/3 group were older (*P* < 0.001; Student's* t-*test) and had a greater number of pregnancies (*P* < 0.001; Mann-Whitney test), and number of childbirths (*P* < 0.001; Mann-Whitney test) than patients from the GWs group. Patients from the CIN 2/3 group also had higher family income (*P* = 0.002; chi-square test), lower monthly frequency of sexual intercourse (*P* = 0.028; Student's *t*-test), and made less use of condoms in the current relationship (*P* = 0.005; chi-square test), compared to patients from the GW group.

Mean treatment duration reported by 60 patients was 17.7 ± 25.2 months, ranging from 1 to 120 months. In the group of patients with GWs, the length of treatment ranged from 1 to 12 months, with a mean of 4.1 months. After this period, the patients no longer considered follow-up visits as “length of treatment.” For women in the CIN group, this time ranged from 2 to 120 months, with a mean of 28.8 months (including the time of followup after conization). The mean number of consultations required to reach a diagnosis reported by 69 patients was 3.3 ± 1.6, ranging from 1 to 6 visits. Of the 75 patients, 34 (45.3%) had to be absent from work in order to undergo treatment. The mean number of absences reported by the 24 patients was 9.3 ± 12.1, ranging from 1 to 48 absences.

The classification of sexual quotient obtained through application of the SQ-F is given in Table 1. It is noteworthy that patients with CIN 2/3 had statistically similar classification of sexual quotient to patients with GWs (*P* = 0.115; chi-square test).

As outlined previously, after application of the SQ-F, several questions were put to the subjects intended to assess the impact of the HPV-induced disease on the sexual lives of the patients. The inferential results showed that the CIN 2/3 patients had statistically similar response profiles compared with GW patients for the following aspects (chi-square test): “Have you told your partner about your disease?” (*P* = 0.569); “Did the relationship break down following your diagnosis?” (*P* = 0.721); “Have you experienced changes in pleasure during sexual intercourse?” (*P* = 0.090); “Has there been a reduction in orgasm since your diagnosis?” (*P* = 0.441); “Has the family dynamic changed due to concerns of infecting family members with the disease (children or household members)?” (*P* = 0.056) (Table 2).

Patients with GWs more frequently gave positive answers (yes) compared to patients with CIN 2/3 (chi-square test) on the following questions: “Has your condition impaired your sexual live in general?” (*P* = 0.020); “Has there been conflict because of a partner attributing the disease to infidelity?” (*P* = 0.024); “Are you now using condoms for all acts of intercourse?” (*P* = 0.040); “Has there been a reduction in your sex drive since your diagnosis?” (*P* = 0.014); “Has the frequency of sexual intercourse changed since your diagnosis?” (*P* = 0.007) (Table 2).  Table 3 presents a summary of the results of comparisons between the CIN 2/3 and GW groups on the relevant variables assessed in this study.

In order to compare the quality (sexual quotient, SQ-F) and impact of disease in sexual life (10 questions applied) of patients with cervical lesions CIN 2/3 and with genital wart, we used the statistical technique of logistic regression (Table 4). The inferential results confirmed that patients with cervical lesions CIN 2/3 have statistically the same rating of sexual quotient when compared to patients with genital warts (*P* = 0.153; logistic regression). 

Considering the impact on sexual life, we noted that patients with cervical lesions CIN 2/3 have statistically the same response profile of patients with genital warts for aspects (by logistic regression): “Have you told your partner about your disease?” (*P* = 0.286); “Has your condition impaired your sexual live in general?” (*P* = 0.165); “Has there been conflict because of a partner attributing the disease to infidelity?” (*P* = 0.502); “Did the relationship break down following your diagnosis?” (*P* = 0.580); “Has there been a reduction in your sex drive since your diagnosis?” (*P* = 0.967); “Have you experienced changes in pleasure during sexual intercourse?” (*P* = 0.804); “Has there been a reduction in orgasm since your diagnosis?” (*P* = 0.575); “Has the family dynamic changed due to concerns of infecting family members with the disease?” (*P* = 0.372).

Patients with cervical lesions CIN 2/3 show positive responses (yes) less frequently when compared to patients with genital warts to aspects: “Are you now using condoms for all acts of intercourse?” (*P* = 0.045); “Has the frequency of sexual intercourse changed since your diagnosis?” (*P* = 0.010).

## 4. Discussion

Despite HPV infection being among the most common STDs seen in clinical practice, attention has only begun to focus on the psychological or psychosexual impact of this diagnosis on the individual. The evidence emerging from the literature and from our clinical experience suggests the existence of several levels of vulnerability because of HPV infection, including impact of the diagnosis, the treatment, and followup. Different stages in these levels may have a different impact on women and couples [7]. 

It is observed that testing positive for HPV and having a visible lesion or a cytological abnormality may have an adverse psychosocial impact, with increased anxiety, distress, and concern about sexual relationships. Negative feelings included fear and anxiety about cancer and becoming ill, concerns about fertility, feelings of being unclean because of the sexually transmitted nature of HPV, concerns about transmission and sexual relationships, a negative impact on feelings about sex, and relationship issues including blaming a partner for the infection. The risk of disease progression has also an impact on sexual health [7, 9].

HPV genital lesions' treatment may be long and painful and can cause sexual impairments. Worry associated with repeated exams and consultations and invasive and painful treatments, which increase in case of recurrences, adds further vulnerability to the woman's emotional and sexual well-being. The higher the number of the interventions, the more painful the technique and the severity of the scarring, the more severe the potential psychosexual impact is. In addition, early and late recurrences of the infection and related pathologies are frequent. They may have a very different impact from the psychosexual point of view, according to the severity of lesions, aggressiveness of related treatments and their side effects, frequency of recurrences and their severity, and quality of psychosexual support from relatives and health care providers [6, 7].

Patients in the present study, when asked about their reaction to receiving the diagnosis of HPV, reported depressive pictures and feelings of indignation, as they believed the disease would remove them from social life and make them a target of prejudice. The partner's reaction to diagnosis disclosure varied from positive attitudes to feelings of blame. It is natural that women with a sole sexual partner who test negative for HPV have questions, concerns, and doubts regarding transmission. 

In this study involving 75 patients, 46 (61.3%) had a histological diagnosis of CIN 2/3 and 29 (38.7%) of GWs. The inferential results indicated that both groups had statistically equal profiles in terms of marital status, smoking habit, age at commencement of sexual activity, number of partners, and use of contraceptive method. Patients in the CIN 2/3 group were generally older and had a greater number of pregnancies and childbirths than patients from the GW group. 

These findings for age and pregnancies corroborate results reported in the literature. Warts associated with low risk viruses trigger the emergence of condylomatous lesions within a short space of time and occur more commonly in young women with lower parity. With regard to CIN, given the natural history of the disease, these tend to manifest later in life, occurring in older women with a greater number of children [10, 11].

An important finding of this study concerned condom use is as follows: although aware of this preventive measure, many patients chose not to make use of condoms; of the 74 patients assessed, 38 (51.4%) did not use condoms. Over time, 26 (36.1%) had stopped using condoms, 19 of whom were influenced by their partners in this decision, evidencing the difficulties and barriers involved in behavioral changes where sex is concerned. 

With regard to the sexual aspect, it is noteworthy that patients with cervical CIN 2/3 had statistically similar classification of sexual quotient compared to patients with GWs. After application of the SQ-F questionnaire, several questions were put to the subjects intended to assess the impact of the disease on the sexual lives of the patients. Patients with GWs more frequently gave positive answers (yes) compared to patients with CIN 2/3 on the following questions: (i) “Has your condition impaired your sexual live in general?”; (ii) “Has there been conflict because of a partner attributing the disease to infidelity?”; (iii) “Are you now using condoms for all acts of intercourse?”; (iv) “Has there been a reduction in your sex drive since your diagnosis?”; (v) “Has the frequency of sexual intercourse changed since your diagnosis?”.

The application of the SQ-F questionnaire suggested a significant impact on the quality of sexual performance among patients. Patients with GWs, however, appear to be more clustered around more favorable categories, whereas individuals with cervical lesions showed a broader distribution across categories, with some cases of zero or poor performance. This phenomenon may be related to the post-surgical period after removal of CIN 2/3 lesions by cervical conization and the subsequent sexual abstinence for a period of at least 45 days. 

The supplementary questions to the SQ-F, however, were more revealing in showing repercussions on the sexual life of the women. Results show that a large proportion of these patients experienced adverse changes in their sexual lives, albeit in sexual intercourse itself, or regarding issues concerning the relationship such as suspected infidelity or breakdown of the relationship.

The few studies that exist suggest that adverse psychological and psychosexual sequelae, related to diagnosis and treatment of lesions associated with HPV infection, may be common. Among these works we can mention Maggino et al. [9], Marra et al. [12], McCaffery et al. [13], Wang et al. [14], and Sénécal et al [15]. However, no study has compared the impact caused by the occurrence of warts and CIN.

Clinical experience indicates that women with a satisfying sexuality before the HPV diagnosis are those less vulnerable to the long-term negative consequences of GWs and their treatments. Vulnerability increases in women experiencing dysfunctional sexuality prior to diagnosis, in single women, in women with troubled relationships or when the infection strongly suggests the partner has had unprotected sex outside of the relationship. Clinical correlates include loss of sexual desire, more difficult mental and genital arousal, dyspareunia, less frequent intercourse, and a qualitative and quantitative reduction of the repertoire of sexual behaviors. After HPV genital infection, many women refuse further passive oral sex for fear of infecting their partner [7]. 

Psychosexual and informative counseling to both partners is critical to prevent further negative psychosexual outcomes during diagnosis and treatment of HPV-related lesions. Husbands and couples express their relief and gratitude when these issues and potential difficulties and/or misunderstandings are openly and spontaneously raised by the physician during the consultation and when practical suggestions are given to overcome physical and emotional problems. Guilty feelings may be pervasive, rooted in the past personal sex life. On the other hand, aggressive feelings against the partner considered responsible for the infection (of having “caught” it) and the subsequent precancerous or cancerous lesions may dominate the clinical picture in a minority of cases. Individual and couple counseling is critical to addressing these feelings that may affect the motivational-affective roots of desire and couple commitment [7, 13]. 

Based on these findings, it is clear that GWs have a greater impact on sexual behavior compared to CIN 2/3. This effect may be explained by the taboo over the presence of GWs, which are highly visible lesions causing symptoms that are characterized as an STD. By contrast, CIN is internal, occult lesions, not regarded as an STD. The increased access to information, particularly on the internet, can propagate myths and fantasies among infected women. 

## Figures and Tables

**Table 1:** Distribution of aspects observed on SQ-F among CIN 2/3 and genital wart patients.

Sexual quotient	CIN 2/3		Genital warts		Total	
Good to excellent	9	19.6%	2	6.9%	11	14.7%
Regular to good	12	26.1%	15	51.7%	27	36.0%
Unfavorable to regular	16	34.8%	8	27.6%	24	32.0%
Poor to unfavorable	3	6.5%	3	10.3%	6	8.0%
Null to poor	6	13.0%	1	3.4%	7	9.3%

Total	46	100.0%	29	100.0%	75	100.0%

Chi-square test.

**Table 2:** Distribution of aspects related to impact of the disease on the sexual life of CIN 2/3 and genital wart patients.

Impact on sexual Life	CIN 2/3		Genital warts		Total	
Told partner^a^						
No	13	28.3%	10	34.5%	23	30.7%
Yes	33	71.7%	19	65.5%	52	69.3%
Total	**46**	**100.0%**	**29**	**100.0%**	**75**	**100.0%**
Caused impairment^b^						
No	27	58.7%	9	31.0%	36	48.0%
Yes	19	41.3%	20	69.0%	39	52.0%
Total	**46**	**100.0%**	**29**	**100.0%**	**75**	**100.0%**
Suspected infidelity^c^						
No	34	73.9%	14	48.3%	48	64.0%
Yes	12	26.1%	15	51.7%	27	36.0%
Total	**46**	**100.0%**	**29**	**100.0%**	**75**	**100.0%**
Relationship breakdown^d^						
No	38	82.6%	23	79.3%	61	81.3%
Yes	8	17.4%	6	20.7%	14	18.7%
Total	**46**	**100.0%**	**29**	**100.0%**	**75**	**100.0%**
Condom^e^						
No	37	80.4%	17	58.6%	54	72.0%
Yes	9	19.6%	12	41.4%	21	28.0%
Total	**46**	**100.0%**	**29**	**100.0%**	**75**	**100.0%**
Reduced sex drive^f^						
No	26	56.5%	8	27.6%	34	45.3%
Yes	20	43.5%	21	72.4%	41	54.7%
Total	**46**	**100.0%**	**29**	**100.0%**	**75**	**100.0%**
Change in frequency^g^						
No	22	47.8%	5	17.2%	27	36.0%
Yes	24	52.2%	24	82.8%	48	64.0%
Total	**46**	**100.0%**	**29**	**100.0%**	**75**	**100.0%**
Change in pleasure^h^						
No	29	63.0%	12	42.9%	41	55.4%
Yes	17	37.0%	16	57.1%	33	44.6%
Total	**46**	**100.0%**	**28**	**100.0%**	**74**	**100.0%**
Reduced orgasm^i^						
No	31	67.4%	17	58.6%	48	64.0%
Yes	15	32.6%	12	41.4%	27	36.0%
Total	**46**	**100.0%**	**29**	**100.0%**	**75**	**100.0%**
Family dynamic^j^						
Sometimes	2	4.4%	2	6.9%	4	5.4%
No	34	75.6%	14	48.3%	48	64.9%
Yes	9	20.0%	13	44.8%	22	29.7%
Total	**45**	**100.0%**	**29**	**100.0%**	**74**	**100.0%**

^a^Have you told your partner about your disease? ^b^Has your condition impaired your sexual live in general? ^c^Has there been conflict because of a partner attributing the disease to infidelity? ^d^Did the relationship break down following your diagnosis? ^e^Are you now using condoms for all acts of intercourse? ^f^Has there been a reduction in your sex drive since your diagnosis? ^g^Has the frequency of sexual intercourse changed since your diagnosis? ^h^Have you experienced changes in pleasure during sexual intercourse? ^i^Has there been a reduction in orgasm since your diagnosis? ^j^Has the family dynamic changed due to concerns of infecting family members with the disease (children or household members)?

Chi-square test.

**Table 3:** Results of comparisons between CIN 2/3 and genital wart groups for relevant variables assessed.

	Conclusion	*P*
Sexual quotient on SQ-F	CIN2/3 = genital warts	0.115
Impact on sexual life (questions)		
Told partner^a^	CIN 2/3 = genital warts	0.569
Caused impairment^b^	CIN 2/3 < genital warts	0.020
Suspected infidelity^c^	CIN 2/3 < genital warts	0.024
Relationship breakdown^d^	CIN 2/3 = genital warts	0.721
Condom^e^	CIN 2/3 < genital warts	0.040
Reduced sex drive^f^	CIN 2/3 < genital warts	0.014
Change in frequency^g^	CIN 2/3 < genital warts	0.007
Change in pleasure^h^	CIN 2/3 = genital warts	0.090
Reduced orgasm^i^	CIN 2/3 = genital warts	0.441
Family dynamic^j^	CIN 2/3 = genital warts	0.056

^a^Have you told your partner about your disease? ^b^Has your condition impaired your sexual live in general? ^c^Has there been conflict because of a partner attributing the disease to infidelity? ^d^Did the relationship break down following your diagnosis? ^e^Are you now using condoms for all acts of intercourse? ^f^Has there been a reduction in your sex drive since your diagnosis? ^g^Has the frequency of sexual intercourse changed since your diagnosis? ^h^Have you experienced changes in pleasure during sexual intercourse? ^i^Has there been a reduction in orgasm since your diagnosis? ^j^Has the family dynamic changed due to concerns of infecting family members with the disease (children or household members)?

Chi-square test.

**Table 4:** Estimates of the parameters of the final logistic regression model.

	*B*	S.E.	Wald	df	Sig.	Exp(*B*)	95% C.I. for Exp(*B*)	
Condom use	1.137	0.566	4.030	1	0.045	3.116	1.027	9.452
Frequency of sexual intercourse	1.534	0.593	6.683	1	0.010	4.636	1.449	14.833
Constant	−1.855	0.555	11.165	1	0.001	0.156		
